# Effect of Manufacture-Induced Interfaces on the Tensile Properties of 3D Printed Polyamide and Short Carbon Fibre-Reinforced Polyamide Composites

**DOI:** 10.3390/polym15030773

**Published:** 2023-02-02

**Authors:** Yingwei Hou, Ajit Panesar

**Affiliations:** Department of Aeronautics, Imperial College London, London SW7 2AZ, UK

**Keywords:** additive manufacturing, defects, short fibre-reinforced polymer, tensile properties

## Abstract

This study aims to elucidate the structure–property–process relationship of 3D printed polyamide and short carbon fibre-reinforced polyamide composites. The macroscopic properties (tensile modulus) of the 3D printed samples are quantitatively correlated to the printing process-induced intrinsic microstructure with multiple interfaces. The samples were printed with different layer thicknesses (0.1, 0.125 and 0.2 mm) to obtain the varied number of interface densities (number of interfaces per unit sample thickness). The result shows that the printed short carbon fibre-reinforced polyamide composites had inferior partially bonded interfaces compared to the printed polyamide, and consequently exhibited interface-dependent elastic performance. The tensile modulus of 3 mm thick composites decreased up to 18% as a function of interface density, whilst the other influencing aspects including porosity, crystallinity and fibre volume fraction (9%) were the same. Injection moulding was also employed to fabricate samples without induced interfaces, and their tensile properties were used as a benchmark. Predictions based on the shear-lag model were in close agreement (<5%) with the experimental data for the injection-moulded composites, whereas the tensile modulus of the printed composites was up to 38% lower than the predicted modulus due to the partial bonded interfaces.

## 1. Introduction

Additive manufacturing (AM), also known as 3D printing, is a fabrication process which adds materials through a successive deposition method (layer by layer) until a final 3D object is fabricated. Compared to traditional subtractive manufacturing methods, AM technology has advantages such as negligible material wastage and manufacturing complex structures without using a mould or assemblies [1]. This leads to significant cost-saving, and therefore AM technology attracts increasing interest from the aerospace [2] and automotive sectors [3].

The material extrusion (ME) method is one of the most-used AM technologies due to its simplicity, and especially recent developments in materials and printers. Available material for ME is mainly thermoplastics such as polylactide (PLA) [4,5,6,7], acrylonitrile butadiene styrene (ABS) [8,9,10,11], polycarbonate [12,13,14] and polyamide [15,16,17]. However, the mechanical performance of the ME-printed thermoplastics shown in Table 1 may not be satisfactory for structural parts. Specifically, the highest tensile strength and tensile modulus of ME-printed PLA reported so far are 40–62 MPa and 3.4~4.7 GPa, respectively. The ME-printed ABS also has a relatively low maximum tensile strength (20~35 MPa) and tensile modulus (1.8~2.2 GPa). The tensile modulus of ME-printed polyamide is up to 0.9 GPa. The unsatisfactory mechanical performance may limit the wide application of ME-printed parts as structural parts in the industry.

Fibre reinforcement is an effective approach to alleviate the drawback of ME-printed thermoplastic applied in the industry. Recent developments in available materials for ME enabled the printing of carbon nanotube [29,30], graphite [31,32], short carbon fibre [19,33,34,35], short glass fibre [36,37], short basalt fibre [38,39] and continuous fibre [40,41,42,43]- reinforced polymer composites. The fibre reinforcements significantly improve the mechanical properties of ME-printed parts. The improvements reported in the literature are summarised in Table 2. For example, the tensile strengths of PLA and ABS with the inclusion of the short carbon fibre reinforcement are seen to improve by up to almost 220% and 240%, respectively. The tensile modulus of continuous carbon fibre-reinforced polyamide is more than an order of magnitude higher than that of the polyamide matrix.

Although the mechanical performance of ME-printed fibre-reinforced polymers (FRPs) is improved, it is not comparable to that of FRPs manufactured via the traditional process, e.g., autoclaves [52]. Firstly, the fibre fraction is relatively low as it is limited by the ME process. The reported maximum fibre contents of short carbon fibre and continuous fibre are 30–40 wt% [45,53] and 50 wt% [21,48], respectively. Increasing fibre content increases the viscosity of the composite, typically leading to nozzle clog interrupting the printing process [45,54,55]. Furthermore, manufacturing defects such as voids may result in high porosity and partial bonded filaments. The porosity of ME-printed FRPs reported so far varies from 7% [56] to 22% [41], and the voids may cause premature failure. Ferreira et al. [33] found noticeable voids in printed short fibred carbon fibre-reinforced PLA and the tensile strength was 53.4 MPa, which was similar to that of pure PLA (54.7 MPa). Zhang et al. [57] found that the tensile strength of short carbon fibre-reinforced ABS was even lower than that of the matrix due to the high porosity. Furthermore, the partial bonded interfaces due to the imperfect coalescence of adjacent filaments may affect the strain distribution of a single layer. Increased strain was found by Christensen et al. [58] at the interfaces between adjacent filaments of transversely printed single-layer sodium alginate relative to the tensile load direction.

The porosity or the partial bonded interfaces may be sensitive to ME parameters such as nozzle temperature and printing speed [59,60,61]. The Markforged^©^ series are provided with an optimised ME process for specific materials, while limiting users’ access to ME parameters. Therefore, the printing process is stable, and the quality of fabricated samples is consistent. Due to this advantage, the Markforged company has an estimated USD 2.1 billion value, and their desktops printers have been adopted widely by the industry [62]. Despite the limited access to ME parameters, there are several options for layer thickness which determine the number of layers printed for a certain sample thickness. As interfaces take place between adjacent layers, the number of interfaces of printed samples can also be determined by layer thickness accordingly.

S. Sommacal et al. [63] analysed the microstructure of ME-printed short fibre-reinforced polyether ether ketone using micro-CT scanning. They found voids in the printed samples aligned in rows parallel to the printing direction. The porosity of printed parts ranged from 19% to 21%, which was independent of the printing parameters. The authors also found the internal microstructure, i.e., voids’ distribution, and the number of interfaces between layers were determined by key printing parameters such as layer thickness and printing temperature. However, the relationship between the microstructure and the mechanical properties of ME-printed samples is not well understood. A quantitative investigation is important for designing and predicting the mechanical performance of ME-printed parts with partial bonded interfaces.

In this paper, the relationship between the ME process-induced microstructure and the properties of printed samples is elucidated by investigating the tensile properties of printed (short carbon fibre-reinforced) polyamide with a different number of interfaces. A Markforged desktop, i.e., Mark Two was used to print polyamide and short fibre-reinforced polyamide samples. The structure of the printed samples was obtained by cryofracture and was observed by scanning electron microscopy (SEM). Prior to the tensile test, influencing factors including porosity, crystallinity and fibre volume fraction were measured and compared. Injection moulding was used to fabricate samples without interfaces, and their tensile performance was used as a benchmark in investigating the effect of interfaces.

## 2. Methods

### 2.1. Materials

The materials were purchased from Markforged^©^ and the polyamide (brand name: nylon) was PA6 indicated by the manufacturer datasheet [64]. Polyamide and short fibre-reinforced nylon (SFRN) filaments (brand name: Onyx) of 1.75 mm diameter from one spool were used to print all specimens via a Mark Two (Markforged, Somerville, MA, USA). The fibre volume fraction of the SFRN filament was about 9% determined by the densities of the matrix and composites measured by a He psycnometry (Accupyc II 1340, Micromeritics Ltd., Hexton, UK). The filaments were conditioned in a vacuum oven at 60 °C for at least 24 h prior to printing as the polyamide is sensitive to moisture [65].

### 2.2. Preparation of Dog-Bone Polyamide and SFRN Samples

#### 2.2.1. ME-Printed Dog-Bone Samples

**Figure 1:**
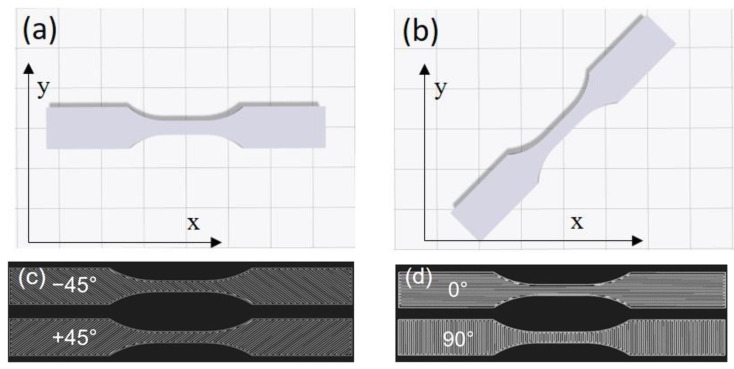
Schematic of sample layouts on the printing platform: (**a**) [+45, −45] and (**b**) [0, 90]. The raster’s arrangement within two consecutive layers: (**c**) [+45, −45] and (**d**) [0, 90].

Tensile test specimens with dog-bone geometry were printed in accordance with the ASTM D638-14 type V [66]. The dimension of the gauge section was 9.53 × 3.18 mm^2^. Consecutive layers were printed in alternating +45 and −45 (+135) degrees relative to the X-axis due to the default printing directions as shown in Figure 1. Specifically, the first layer was printed in +45 degrees, while the second layer was printed in −45 degrees. The specimens were printed in a rectangular infill pattern with 100% infill density, which may provide the highest tensile properties [50]. The printing temperature was pre-set at 275 °C and the default printing speed was estimated to be 17 mm/s. The setting values were kept constant during the printing process for all the specimens.

The above-mentioned printing directions were consistent and set by the printer-control software, i.e., Eiger. However, two raster patterns, i.e., [+45, −45] and [0, 90] relative to the tensile load direction, could be printed by rotating the specimen layout on the printing platform by 0° and 45°, respectively. Figure 1 shows the schematic of the specimen layout in Eiger and the raster patterns within the specimens. Specimens printed with the [+45, −45] and [0, 90] patterns had interfaces at +45/−45 and 0/90 degrees relative to the tensile load direction, respectively.

Layer thickness values were also set by Eiger, and all available options (0.1, 0.125 and 0.2 mm) were chosen to obtain different interface densities, i.e., the number of interfaces per unit sample thickness. The thickness values of samples were 2, 3 and 4 mm to obtain the integer number of total printed layers which equalled the sample thickness divided by layer thickness, and meanwhile the sample thickness was under 4 mm for the type V specimen for the tensile test in accordance with ASTM D638-14. The sample thickness was changed to investigate the effect of the interface density on the tensile properties of printed SFRN with the different total number of layers. The polyamides printed with 0.1, 0.125 and 0.2 mm layer thicknesses were named polyamide_0.1, polyamide_0.125 and polyamide_0.2, respectively. Similarly, the SFRN samples were named SFRN_0.1, SFRN_0.125 and SFRN_0.2.

#### 2.2.2. Injection-Moulded Dog-Bone Samples

Materials from the same spool for the ME-printed samples were also used for injection moulding (HaakeMinijet II, ThermoFisher Scientific, Hampshire, UK). Pellets cut from the spools were melted in a barrel at 260 °C for 120 s prior to injection and then injected into a mould at 80 °C under the pressure of 600 bar for 10 s. A post-pressure of 100 bar was maintained for 60 s after the injection process. The injection-moulded samples were 3 mm thick and had the same geometry and dimensions as the ME-printed samples.

### 2.3. Porosity Measurement of Filaments and Fabricated Samples

The polyamide and the SFRN filaments were cylindrical, and they were cut into segments of about 1 m length. The cross-section diameter (*d*) of the segments (five specimens) was measured using a calliper. The bulk density ($\rho_{1}$) was calculated after weighing the mass (*m*) of the segments following Equation (1):

(1) $$\rho_{1}=\frac{m}{v}=\frac{m}{1\times \pi \times {\left(\frac{d}{2}\right)}^{2}}$$

The bulk density of fabricated samples for the tensile test was measured by dividing the weighed sample mass by their envelope volume, which was evaluated by a computer-aided design software (SolidWorks^®^ 2018). The density of pellets cut from the spools using a pelletizer machine (VariCut Pelletizer, Thermo Fisher Scientific Inc., Waltham, MA, USA) was measured by He pycnometry (Accupyc II 1340, Micromeritics Ltd., Hexton, UK). The pellets were assumed to have no voids, and their density referred to the true density ($\rho_{t}$) of the materials. The porosity (P) of the samples was measured due to the difference between the measured bulk densities $\left(\rho_{b}\right)$ and the true density. The measurement was conducted as follows:

(2) $$P\;\left[\%\right]=\left(1-\frac{\rho_{b}}{\rho_{t}}\right)\times 100$$

### 2.4. Crystallinity of Filaments and Fabricated Samples

The crystallinity and the melting temperature of the filaments and the fabricated samples for the tensile tests were measured by differential scanning calorimetry (DSC, Discovery DSC, TA Instruments, Newcastle, UK). Samples consisting of five specimens with about 3–5 mg were heated in a nitrogen atmosphere from 20 °C to 275 °C, and then cooled to 20 °C prior to a second heating to 275 °C. Both the heating and the cooling rates were 5 °C min^−1^. The crystallinity ($\chi_{c}$) was measured as below:

(3) $$\chi_{c}=\frac{\Delta H_{m}}{\left(1-\alpha \right)\Delta H^{\emptyset }}$$

where $\Delta H_{m}$ refers to the melting enthalpy of the samples measured by calculating the area under the endothermic peak shown on the heating curves. $\Delta H^{\emptyset }$ denotes the melting enthalpy of pure polyamide, taken as 230 J/g [65]. The symbol α refers to the mass fraction of carbon fibre, and it was taken as zero for the polyamide samples.

### 2.5. Tensile Properties of Fabricated Polyamide and SFRN

Tensile tests on the fabricated dog-bone samples were conducted on an Instron universal machine (Model 5960, Instron, Norwood, MA, USA) equipped with a 10 kN load cell following the ASTM D638-14. A speckle pattern was applied to the gauge section of the specimens using an ink stamp. The strain of the specimens subjected to 10 mm min^−1^ displacement was measured by monitoring the patterns’ movement using a non-contact video extensometer (iMetrum Video Gauge, Bristol, UK). Each sample consisted of five specimens, and the tensile test was repeated twice for each sample.

### 2.6. Shear-Lag Model

The tensile properties of the short fibre-reinforced composites can be described by the shear-lag theory developed by Cox and Krenchel [67,68]. The assumptions of the theory are as follows: (1) both fibre and matrix deform elastically, (2) the fibre/matrix interface is intact and (3) no load at the fibre ends. The shear-lag theory generally underestimates the stiffness of short fibre composites as it neglects stress at the fibre ends [69]. The predicted tensile modulus ($E_{c}$) of composites based on the shear-lag model is given by

(4) $$E_{c}={ɳ}_{0}{ɳ}_{L}V_{f}E_{f}+\left(1-V_{f}\right)E_{m}$$

where $E_{m}$ and $E_{f}$ refer to the tensile modulus of matrix and fibre, respectively. $V_{f}$ refers to the fibre volume fraction of composites. ${ɳ}_{L}$ is a length correction factor due to the finite length of fibre written as

(5) $${ɳ}_{L}=1-\frac{\tanh\left(\frac{\beta L}{2d}\right)}{\frac{\beta L}{2d}}$$

(6) $$\beta =\sqrt{\frac{2E_{m}}{E_{f}\left(1+\nu_{m}\right)\ln\left(\frac{1}{V_{f}}\right)}}$$

where *L* and *d* refer to the length and the diameter of the fibre, respectively. $\nu_{m}$ is the Poisson’s ratio of matrix. ${ɳ}_{0}$ is a fibre-orientation factor referring to the fraction of fibre ($V_{f,\;n}$) aligning at angle $\theta_{n}$ relative to the tensile load written as

(7) $${ɳ}_{0}=\frac{\sum_{n}V_{f,\;n}\cos^{4}\theta_{n}}{\sum_{n}V_{f,\;n}}$$

### 2.7. Structure Morphology of Fabricated Polyamide and SFRN

The fabricated samples were cryofractured by placing them in liquid nitrogen for 10 min, and then the cross-section structure was observed by scanning electron microscopy (SEM, Hitachi S-3700N, Tokyo, Japan). The samples were firstly mounted onto aluminum stubs with carbon tabs and then coated with Au (Agar Auto Sputter Coater, Essex, UK) with a coating current of 40 mA for 60 s. The short carbon fibre in SFRN filaments was observed by optical microscope (Axioscope, Zeiss, Germany), and its dimension was measured by ImageJ software (Rasband, W.S., ImageJ, USA).

## 3. Results and Discussion

### 3.1. Thermal Properties of Filaments and Fabricated Samples

**Figure 2:**
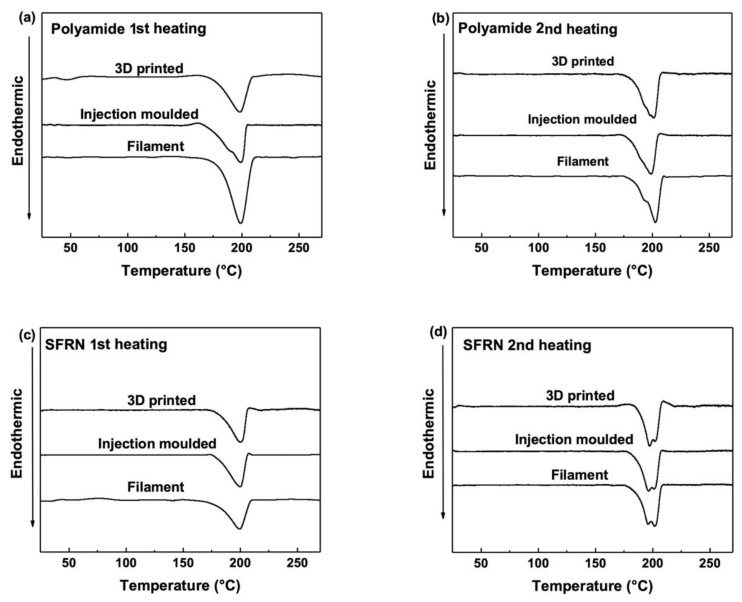
DSC curves of polyamide during (**a**) first heating and (**b**) second heating; SFRN composites during (**c**) first heating and (**d**) second heating.

The crystallinity of the filaments and the fabricated samples was measured based on the DSC curves shown in Figure 2. The DSC results for the ME-printed samples are shown by a representative curve for simplicity, as the DSC curves of the printed samples are similar. All the heating curves show an endothermic peak corresponding to the melting process of polyamide. The peak point is about 200 °C referring to the melting temperature of the samples, and the value is close to the results from the literature for polyamide 6 [65,70].

Table 3 summarises the crystallinity and the melting temperature (T_m_) of the filaments and the fabricated samples based on the heating curves. Both the ME-printed samples and the injection-moulded samples have similar crystallinity. Specifically, the crystallinity of the ME-printed samples based on the 1st heating curves is 16–19%, whereas that of the injection-moulded samples is 18%. Furthermore, the polyamide filaments have 35% crystallinity, which is higher than that of the printed polyamide. The ME process is a rapid manufacturing process and the printed samples may cool down to ambient temperature in seconds [71], resulting in insufficient time for crystallisation. After the thermal history induced by the manufacturing process was removed by the 1st heating, the printed samples had similar crystallinity as that of filaments on the 2nd heating curves.

The first heating curves reveal the crystallinity determined by the manufacturing process. The similar crystallinity of the printed samples indicates the crystallisation is not influenced by the raster patterns and the layer thickness. All the ME-printed samples and the injection-moulded samples have similar crystallinity, indicating that the factor of crystallinity would not result in any variation in the tensile properties of the fabricated samples.

### 3.2. The Structure of the Fabricated Samples

Firstly, the porosity of the polyamide and the SFRN filaments is 2–3%, while the printed polyamide and the SFRN samples have the porosities of 5% and 10%, respectively. The higher porosity indicates the printing process may induce extra voids in the printed samples. Therefore, the structure of the printed samples was analysed to investigate the voids induced by the printing process.

**Figure 3:**
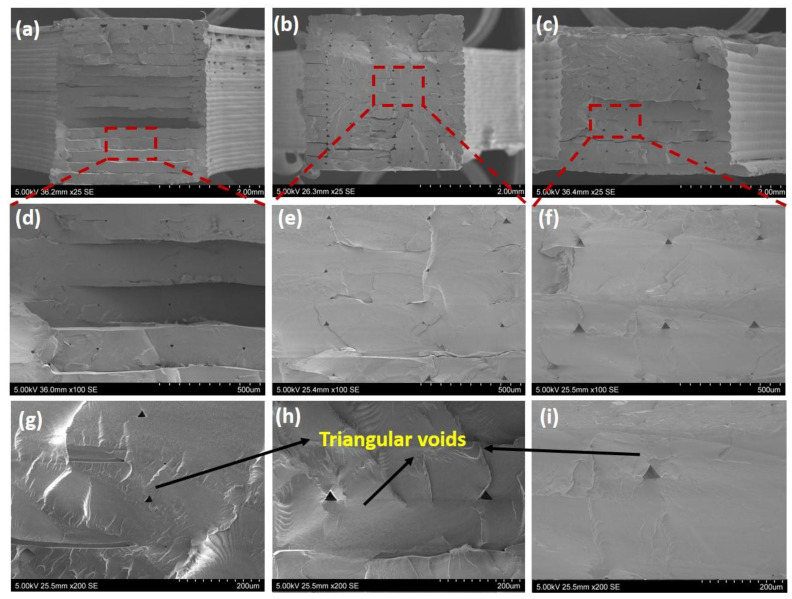
The structure of the printed [0, 90] polyamide samples at different magnifications: 25×: (**a**) polyamide_0.1, (**b**) polyamide_0.125, (**c**) polyamide_0.2; 100×: (**d**) polyamide_0.1, (**e**) polyamide_0.125, (**f**) polyamide_0.2; 200×: (**g**) polyamide_0.1, (**h**) polyamide_0.125, (**i**) polyamide_0.2.

Figure 3 shows the cryofracture surface of the printed 3 mm thick [0, 90] polyamide samples with 0.1, 0.125 and 0.2 mm layer thicknesses. Triangular voids with 29 µm to 47 µm edge length were found between adjacent filaments due to their partial coalescence. The reason for this incomplete fusion of filaments is the rapid cooling and solidification of the extruded materials. Therefore, the printed samples have partial bonded interfaces between filaments induced by the manufacturing process. The partial bonded interfaces may exist along the filament’s longitudinal direction, thereby resulting in micro-size channels. The number of the interfaces is a function of the number of printed layers, which is determined by layer thickness. Specifically, 0.1 mm layer thickness has the greatest number of interfaces when compared to 0.125 or 0.2 mm layer thickness for the same sample thickness, leading to the greatest interface density.

**Figure 4:**
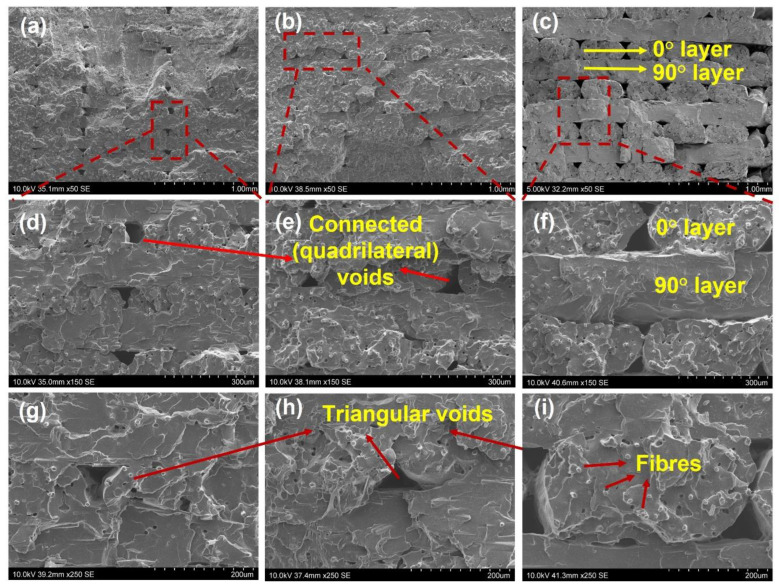
The structure of the printed [0, 90] composite samples with different layer thickness: 50×: (**a**) 0.1 mm, (**b**) 0.125 mm, (**c**) 0.2 mm; 150×: (**d**) 0.1 mm, (**e**) 0.125 mm, (**f**) 0.2 mm; 250×: (**g**) 0.1 mm, (**h**) 0.125 mm, (**i**) 0.2 mm.

Compared with the printed polyamide samples, the structure of the printed [0, 90] SFRN samples (see Figure 4) shows large-size voids (edge length: 75~93 µm) taking place between the filaments. Voids are not only triangular in shape but form quadrilateral shapes resulting from the joining of two triangular voids. This indicates the manufacturing process-induced interfaces of the printed SFRN samples are inferior to those of the printed polyamide. Consequently, the printed SFRN samples have higher porosity (10%) than that (5%) of the printed polyamide. The SFRN filaments in 0° direction are more distinguishable due to the inferior interfaces. The number of 0° filaments at the gauge section (width: 3.18 mm) is eight, which is independent of sample thickness and layer thickness. Therefore, interfaces within a layer are consistent, and the interface density is only determined by layer thickness. Furthermore, fibres with round cross-sections were observed in 0° SFRN filaments. This indicates fibres relatively align along filaments’ longitudinal direction (printing direction).

It is worth mentioning that the porosity of the printed SFRN with the same sample thickness is independent of layer thickness (interface density). On the one hand, the lower layer thickness determines the greater number of layers printed within the sample, which may result in the greater number of heating cycles. The thermal conduction from the upper layers can improve the filaments’ fusion of bottomed layers [71,72] and lead to lower porosity of the bottom part [63]. However, the greater number of partial bonded interfaces could take place when more layers are printed.

**Figure 5:**
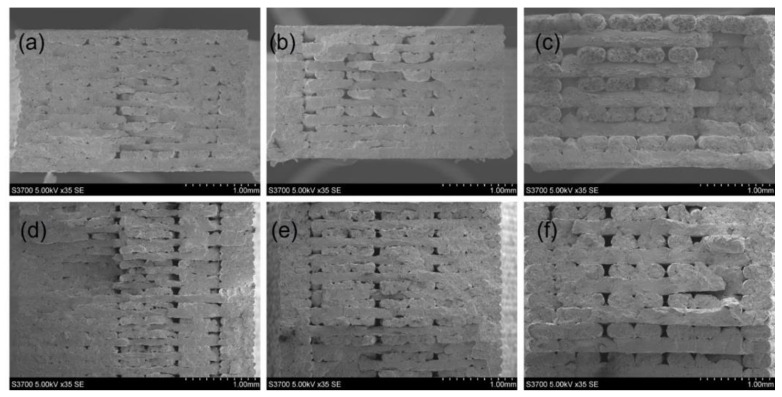
SEM images (35×) of the structure of the printed [0, 90] composite samples with different thickness: 2 mm: (**a**) SFRN_0.1, (**b**) SFRN_0.125, (**c**) SFRN_0.2; 4 mm: (**d**) SFRN_0.1, (**e**) SFRN_0.125, (**f**) SFRN_0.2.

The structure of printed SFRN samples with 2 mm and 4 mm shown in Figure 5 also shows the inferior interfaces in triangular and quadrilateral geometry. The porosity of 2 mm and 4 mm thick samples is 12% and 8%, respectively, which was measured using the method described in Section 2.3. The lower porosity might have resulted from the slower cooling of the 4 mm thick sample, as the cooling rate depends on the sample thickness. The slower cooling rate may lead to the better fusion and coalescence of the printed filaments [71]. The influences of key printing parameters on material fusion and the resulting porosity of printed parts need more research efforts.

**Figure 6:**
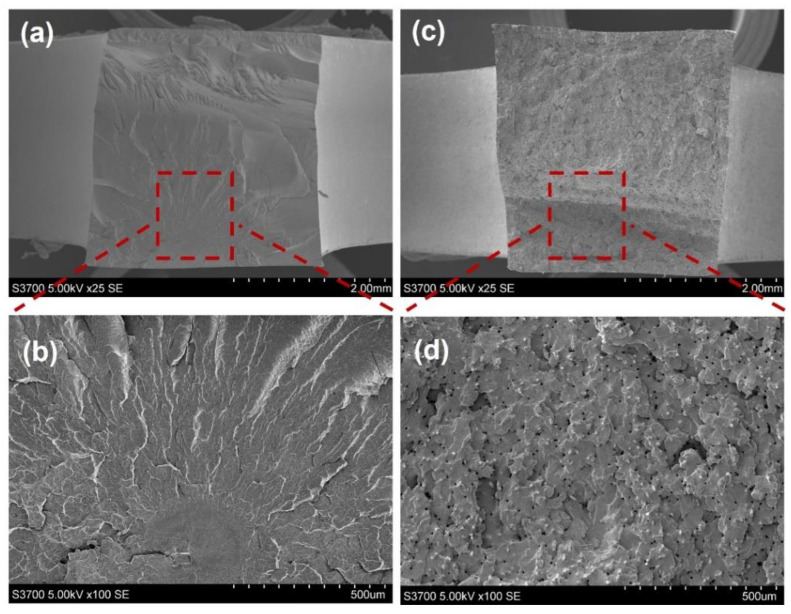
The structure of injection-moulded polyamide: (**a**) 25× and (**b**) 100×; injection-moulded SFRN: (**c**) 25× and (**d**) 100×.

The porosities of 2 mm and 4 mm thick polyamide samples are similar (about 5%). The printed polyamide has superior interfaces due to better filaments’ fusion, and therefore the porosity is less dependent on the sample thickness or layer thickness. The structure of injection-moulded samples (see Figure 6) was also investigated for comparison with the printed samples. Voids as well as the partial bonded interfaces were not found in the samples and the resulting porosity is less than 1%. Therefore, the number of interfaces of injection-moulded samples would be assumed as zero.

### 3.3. The Tensile Properties of the Fabricated Samples

#### 3.3.1. Comparison between the Injection-Moulded Samples and the Printed Samples

**Figure 7:**
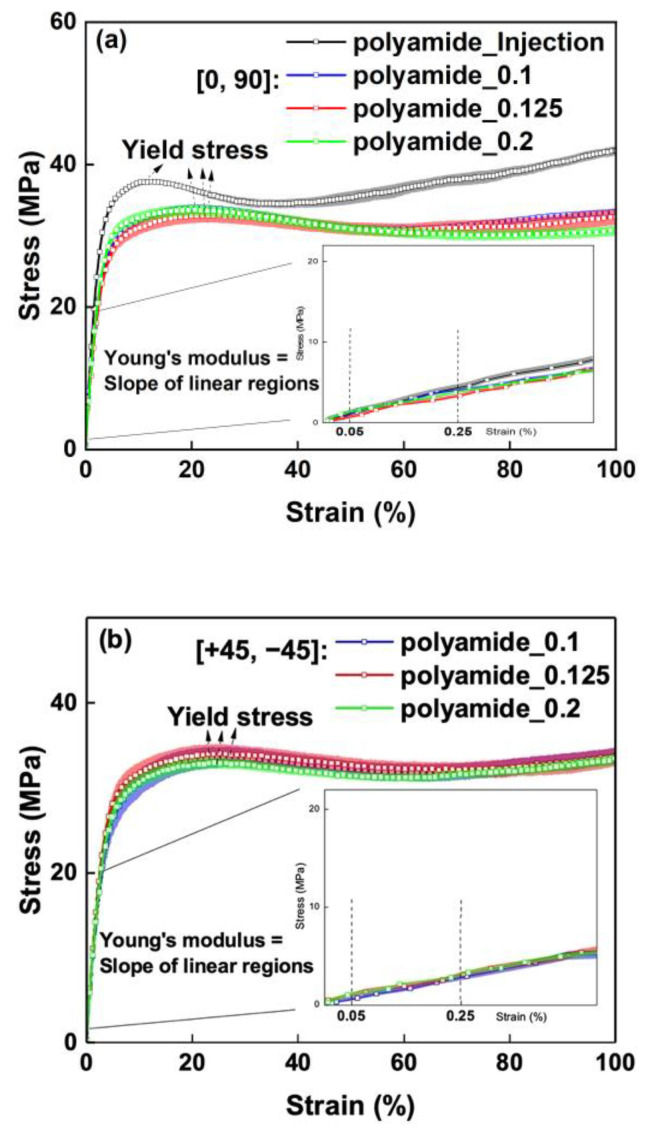

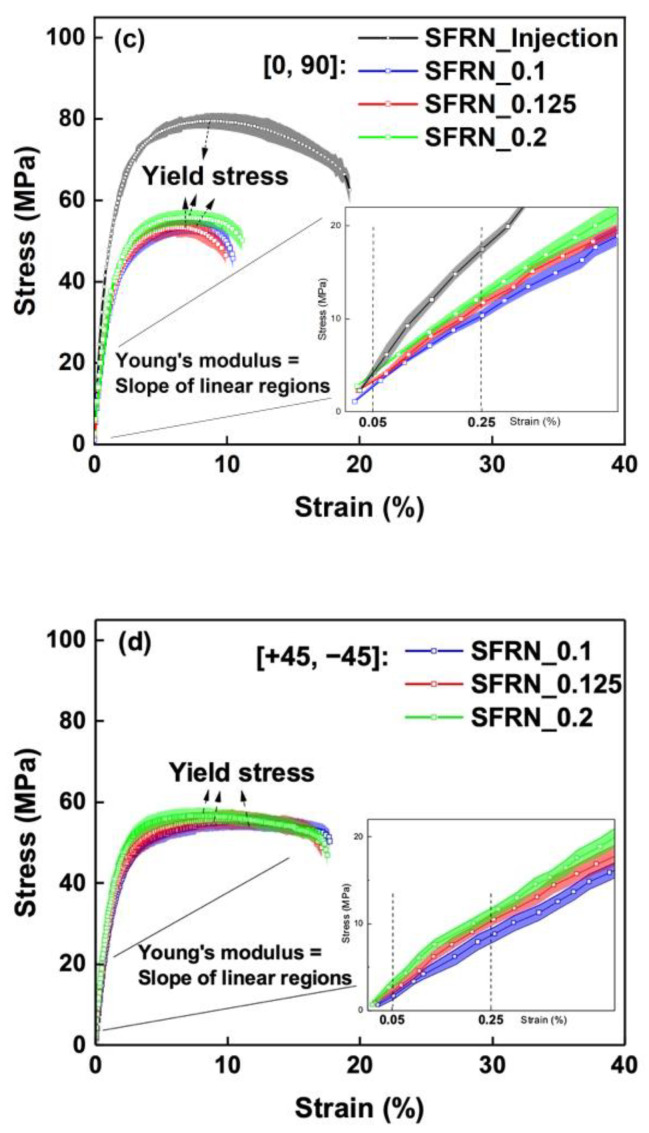
The strain–stress curves of the fabricated samples with 3 mm thick: (**a**) injection-moulded and [0, 90] polyamide; (**b**) [+45, −45] polyamide; (**c**) injection-moulded and [0, 90] SFRN; (**d**) [+45, −45] SFRN.

The stress–strain curves of the fabricated samples with 3 mm thickness are plotted in Figure 7. The standard deviation of each sample (six specimens) is presented by the upper and lower bounds of the stress–strain bands. Strain values obtained from the video extensometer and tensile properties (yield stress and tensile modulus) were determined from the stress–strain curves.

The yield stress is considered as the point on the stress–strain curve where the stress did not increase with increasing strain (zero slope). The yield stress of SFRN refers to yield strength at the yield point, and all SFRN specimens broke at the gauge section area. The tensile modulus is determined by the slope of linear regression lines in the strain range of 0.05% and 0.25%. The measured tensile properties of the fabricated samples are summarised in Table 4.

Firstly, the tensile properties of the injection-moulded samples are compared to the printed samples. The yield stress and the tensile modulus of the injection-moulded polyamide are 37.6 MPa and 1.43 GPa, respectively. The yield stress and the tensile modulus of the printed polyamide are 9–12% and 17–22% lower compared to the injection-moulded sample, respectively. The printed polyamide has higher porosity and the partial bonded interfaces, which may contribute to the lower tensile properties as interfaces could cause non-uniform strain distribution and lead to premature failure. Furthermore, the failure strain of the injection-moulded SFRN is about 20 ± 1%, whereas the printed [0, 90] and [+45, −45] SFRN samples fail at about 10 ± 1% and 17 ± 2%, respectively. The lower failure strain of printed samples indicates premature failure took place due to the induced interfaces. The result also indicates the interfaces in [0, 90] SFRN have a more significant impact on premature failure compared to the interface in [+45, −45] SFRN. The transverse interfaces relative to tensile load direction in the printed [0, 90] may act as cracks leading to stress concentration and premature failure.

The yield stress and the tensile modulus of the injection-moulded SFRN are 78.1 MPa and 6.67 MPa, respectively. Compared to the injection-moulded samples, the yield stress and the tensile modulus are 25–30% and 31–45% lower, respectively. The printed SFRN samples have a more significant reduction in tensile properties than the printed polyamide, due to the inferior partial bonded interfaces. The contact area of adjacent SFRN filaments is reduced by the larger-size interfaces as well as the connected interfaces, resulting in a more substantial decrease in load-transfer efficiency. Therefore, the influence of the partial bonded interfaces is more significant on the tensile properties of the printed SFRN.

Table 4 also summarises the tensile properties of the printed samples with 2 mm and 4 mm thickness. The yield stress and the tensile modulus of the printed polyamide are 30.3–34.1 MPa and 1.11–1.21 GPa, respectively. The tensile properties of the printed polyamide are close and not influenced by sample thickness. However, the tensile modulus of the printed SFRN increases with the sample thickness. For example, the tensile modulus of [0, 90] SFRN_0.2 with 4 mm thickness is 29.6% higher than that of [0, 90] SFRN_0.2 with 2 mm thickness. The reason might be the porosity of the printed SFRN decreasing with the sample thickness. The higher porosity of 2 mm thick SFRN samples may result in the lower tensile modulus.

#### 3.3.2. Shear-Lag Theory Analysis

The shear-lag effect happens in discontinuous fibre composites in which the fibre and polymer matrix have an apparent mismatch in the modulus. According to shear-lag theory, the efficiency of short carbon fibre is limited due to its discontinuity, and axial stress is transferred to fibre by shear stress at the fibre/matrix interface. Consequently, the stiffness of composites degrades due to the inefficiency of short fibre.

**Figure 8:**
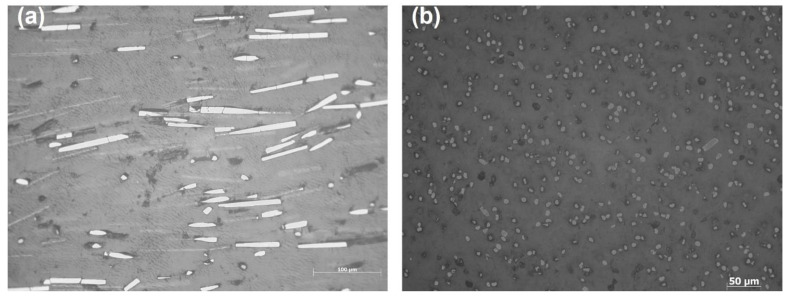
The microscope images of short carbon fibres in SFRN filaments: (**a**) longitudinal; (**b**) axial.

The measured fibre volume fraction of fabricated SFRN is about 9%. The length and the diameter of short fibre are 140 ± 10 µm and 7 ± 0.5 µm based on the microscope images of fibres shown in Figure 8. The ${ɳ}_{0}$ values for printed SFRN and injection SFRN are taken as 0.56 and 0.80 based on the literature [69,73], respectively. The tensile modulus of carbon fibre is 230 GPa [74] and the tensile modulus of polyamide is 1.4 GPa due to the tensile result of the injection-moulded polyamide. The Poisson’s ratio of polyamide is assumed to be 0.39 as suggested in the literature [75].

The predicted tensile modulus of the injection-moulded SFRN based on the shear-lag model is 6.36 GPa, which is in good agreement (<5% difference) with the experimental value (6.67 GPa). This result indicates the loss on the tensile modulus of the fabricated SFRN due to the limited fibre length and the reduced efficiency of fibre reinforcement. However, the predicted tensile modulus (4.84 GPa) is greater than the experimental measured modulus of the printed SFRN, except the 4 mm SFRN_0.2 sample. The tensile modulus of 2 mm [+45, −45] SFRN_0.1 is almost 38% lower than the predicted modulus. The reasons could be the printing-induced partial bonded interfaces in SFRN which are not considered in the theory. Furthermore, the shear-lag effect between the fibre/matrix may not be sufficient in explaining the variation on the tensile modulus of the printed SFRN with multiple partial bonded interfaces.

#### 3.3.3. The Effect of Interfaces on the Tensile Modulus of the Printed Samples

**Figure 9:**
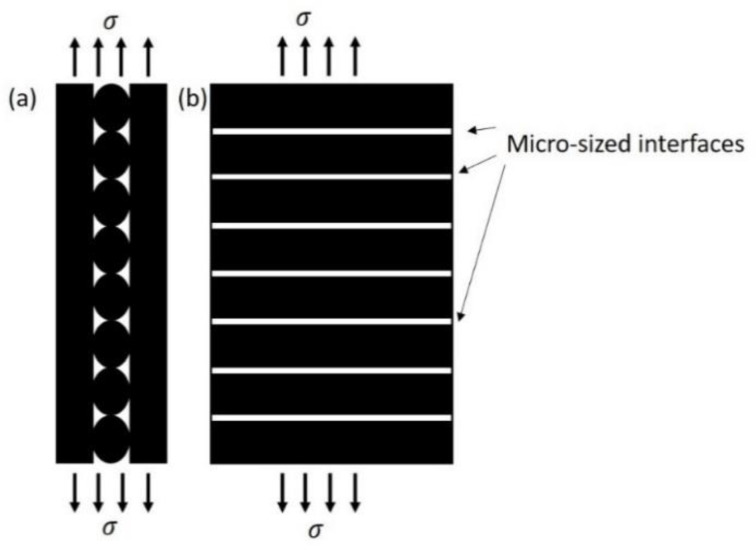
The schematic of transverse interfaces in [0, 90] samples: (**a**) edge view; (**b**) top view.

As discussed above, inferior interfaces with a larger size were found between adjacent SFRN filaments compared to the polyamide, resulting in a more significant reduction in the tensile properties. The partial bonded interfaces may continuously distribute along the filament direction, and therefore the printed [0, 90] and [−45, +45] samples may consist of 90° and 45° interfaces relative to the tensile load, respectively. The load transfer in laminates subject to the tensile load is degraded due to the off-axis pre-existing defects where the load carried by the layers is transferred to the neighbouring layers. The degraded load transfer resulting in the reduction in stiffness is described by various approaches such as shear-lag models [76,77,78], McCartney’s models [79] and crack-opening-displacement models [80]. The schematic of the transverse interfaces in [0, 90] samples is shown in Figure 9. The transverse interfaces may also influence the load transfer of the printed samples subject to tensile load, and then lead to the reduction in the tensile modulus.

The average tensile modulus of the printed polyamide summarised in Table 4 is about 1.15 GPa, which is independent of the layer thickness and raster pattern. However, the tensile modulus of the printed SFRN samples increases with the layer thickness. For example, the tensile modulus of 3 mm thick [0, 90] SFRN_0.1 is 4.9% and 17.8% lower than that of SFRN_0.125 and SFRN_0.2, respectively. The tensile modulus of 2 mm and 4 mm thick SFRN samples also increases with the layer thickness.

**Figure 10:**
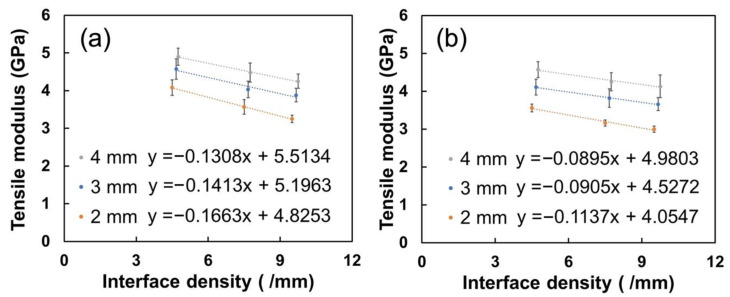
The tensile modulus of the printed SFRN samples as a function of interface density: (**a**): [0, 90] SFRN; (**b**): [+45, −45] SFRN.

Layer thickness determines the number of printed layers for certain sample thicknesses, i.e., more interfaces (higher interface density) may take place. The interfaces may result in the reduction in tensile modulus, and a higher interface density may lead to a more significant reduction. The number of interfaces as well as interface density are summarised in Table 5. The relationship between interface density and tensile modulus is quantitatively correlated in Figure 10. The correlation coefficients are summarised in Table 5.

The correlation suggests that the tensile modulus of the printed SFRN samples decreases linearly with the interface density as described below:

(8) $$E=\alpha \cdot I_{d}+E_{0}$$

where E and $E_{0}$ refer to the tensile modulus of the printed SFRN samples and the tensile modulus of the printed SFRN having no interfaces or porosity, respectively. $I_{d}$ refers to interface density and α is a coefficient. Firstly, the coefficient α for [0, 90] and [+45, −45] SFRN is about −0.16~−0.13 and −0.09~−0.11, respectively. The negative coefficients reveal the interface density has a negative influence on the tensile modulus of the SFRN samples. The [0, 90] SFRN samples have a relatively higher coefficient α compared to [+45, −45]. This indicates the transverse partial bonded interfaces relative to load direction in [0, 90] samples could degrade the load transfer more significantly, resulting in the greater loss of stiffness. Secondly, the y-intercept refers to the tensile modulus ($E_{0}$) of printed samples without interfaces. The $E_{0}$ of [+45, −45] SFRN samples ranges from about 4.1 GPa to 5.0 GPa, which is relatively lower compared to [0, 90] SFRN (4.8 GPa to 5.5 GPa), which may have resulted from the anisotropic performance due to the filament orientations. Fires were found to relatively align along the filaments’ orientation, and therefore the printed SFRN samples may exhibit an anisotropic performance. The tensile modulus of the fibre-reinforced polymer composite with [0, 90] layup sequence has been reported to be higher compared to [−45, +45] layup [74].

It is worthy to mention that the $E_{0}$ of the printed SFRN decreases with sample thickness due to the increasing porosity. The 2, 3 and 4 mm SFRNs have 7%, 10% and 12% void contents, respectively. This also explains that the $E_{0}$ of the printed SFRN is still lower than that of the injection-moulded SFRN (6.67 GPa) as the samples have less than 1% porosity. As the correlations are based on the limited range of interface density, more research work would be helpful to improve accuracy and applicability.

## 4. Conclusions

This study investigates the influence of printing-induced defects at the interface on the tensile properties of (short carbon fibre-reinforced) polyamide. A correlation between the interface density and the tensile properties is analysed and quantified. The main achievements are summarised below:

- Firstly, relying on the commercial Markforged printer with limited access to processing parameters except layer thickness, the printed samples exhibit consistent quality including porosity, crystallinity and fibre volume fraction.
- Secondly, printing process-induced interfaces are found in both printed polyamide and SFRN samples. The partial bonded interfaces are distributed at the interface between printed filaments. The interface density increases when layer thickness decreases from 0.2 mm to 0.1 mm. Compared to the printed polyamide, the printed SFRN samples have inferior interfaces with a larger size.
- Consequently, the tensile properties of the printed SFRN are more significantly lower than those of the injection-moulded SFRN. The printed polyamide exhibits a relatively lower yield stress (9–12%) and tensile modulus (17–22%) compared to the injection-moulded sample, whereas the yield stress and the tensile modulus of the printed SFRN are 25–30% and 31–45% lower, respectively.
- Furthermore, the tensile modulus of the printed SFRN decreases as a function of interface density, while the tensile modulus of the printed polyamide is independent of interface density. The tensile modulus of the 3 mm thick [0, 90] SFRN_0.1 is 4.9% and 17.8% lower compared to SFRN_0.125 and SFRN_0.2, respectively. A shear-lag model is found to predict the tensile modulus in good agreement with the experimentally measured modulus of the injection-moulded SFRN. However, the experimental modulus of the printed SFRN is lower than the predicted modulus due to the printing-induced interfaces in SFRN.
- Lastly, the quantitative correlation between the tensile modulus of the SFRN and the interface density is analysed. An empirical model is developed based on data fitting, and the model shows that the tensile modulus of the printed SFRN decreases with interface density following a linear function. This result suggests that the quantitative degradation of the stiffness due to interfaces should be considered when designing 3D printed parts for engineering applications. The microstructure can be improved to achieve a maximum and interface-independent mechanical performance.

## Figures and Tables

**Table 1:** The mechanical properties of ME-printed thermoplastic polymer reported in the literature.

Authors	Materials	Test Type	Standard	Results *
Song et al. [18]	PLA	Tensile; Compressive	Not reported (NR)	$\sigma_{t}$: 55 MPa; $E_{t}$: 4.0 GPa; $\sigma_{c}$: 98 MPa; $E_{c}$: 4.7 GPa
Yao et al. [4]	PLA	Tensile; Flexural	ISO 527; ISO 14125	$\sigma_{t}$: 46 MPa; $\sigma_{f}$: 82 MPa
Ning et al. [19]	ABS	Tensile	ASTM D638	$\sigma_{t}$: 34 MPa; $E_{t}$: 1.9 GPa
Love et al. [20]	ABS	Tensile	ASTM D638	$\sigma_{t}$: 35 MPa; $E_{t}$: 2.2 GPa
Omuro et al. [21]	PLA	Tensile; Flexural	NR	$\sigma_{t}$: 40 MPa; $E_{t}$: 4.7 GPa; $\sigma_{f}$: 65 MPa;$\;E_{f}$: 2.5 GPa
Tian et al. [22]	PLA	Tensile; Flexural	GB/T 1447; GB/T 1449	$\sigma_{t}$: 62 MPa; $E_{t}$: 4.2 GPa; $\sigma_{f}$: 100 MPa;$\;E_{f}$: 4.0 GPa
Van Der Klift et al. [23]	Polyamide	Tensile	JIS K 7073	$E_{t}$: 0.9 GPa;
Tymrak et al. [8]	ABS; PLA	Tensile	ASTM D638	ABS: $\sigma_{t}$: 29 MPa; $E_{t}$: 1.8 GPa; PLA:$\;\sigma_{t}$: 57 MPa; $E_{t}$: 3.4 GPa;
Cantrell et al. [24]	ABS; PC	Tensile	ASTM D638	ABS: $\sigma_{t}$: 30 MPa; $E_{t}$: 2.0 GPa; PC: $\sigma_{t}$: 54 MPa; $E_{t}$: 1.9 GPa;
McLouth et al. [25]	ABS	Fracture Toughness	ASTM D5045	1.97 MPa m^1/2^
D’Amico et al. [9]	ABS	Tensile; Flexural	ASTM D638; ASTM D790	$\sigma_{t}$: 20 MPa;$\;\sigma_{f}$: 21 MPa;
Rahmatabadi et al. [26]	PLA-polyurethane	Tensile; Compressive; Flexural	ASTM D638; ISO604:2002; ASTM D790	$\sigma_{t}:\;54$ MPa; $\sigma_{c}$: 43 MPa $\sigma_{f}$: 124 MPa;
Rahmatabadi et al. [27]	Poly vinyl chloride	Tensile; Compressive; Flexural	ASTM D638; ISO604:2002; ASTM D790	$\sigma_{t}$: 77 MPa; $E_{t}$: 0.7 GPa $\sigma_{c}$: 57 MPa; $E_{c}$: 0.8 GPa $\sigma_{f}$: 201 MPa; $E_{f}$: 1.3 GPa
Moradi et al. [28]	polyamide	Tensile	ASTM D638	Elongation: 596%

* $\sigma_{t}$, $\sigma_{f}$, $\sigma_{c}$ refer to tensile strength, flexural strength and compressive strength, respectively. $E_{t}$, $E_{f}$, $E_{c}$ refer to tensile modulus, flexural modulus and compressive modulus, respectively.

**Table 2:** The mechanical improvement of fibre-reinforced polymer manufactured via ME.

Authors	FRPs	Fibre Fraction	Test Type	Standard	Mechanical Improvements *
Ning et al. [19]	Short carbon fibre/ABS	5 wt%	Tensile	ASTM D638	$\sigma_{t}$: 24%; $E_{t}$: 32%;
Love et al. [20]	Short carbon fibre/ABS	13 wt%	Tensile	ASTM D638	$\sigma_{t}$: 236%; $E_{t}$: 427%;
Mahajan and Cormier [44]	Short carbon fibre/epoxy	15 wt%	Tensile	ASTM D638	$\sigma_{t}$: 41%; $E_{t}$: 45%;
Omuro et al. [21]	Continuous carbon fibre/PLA	30 vol%	Tensile; Flexural	NR	$\sigma_{t}$: 1389%; $E_{t}$: 1356%; $\sigma_{f}$: 1012%;$\;E_{f}$: 242%
Ferreira et al. [33]	Short carbon fibre/PLA	15 wt%	Tensile; Shear	ASTM D638; ASTM D3518	$\sigma_{t}$: 220%; $\sigma_{s}$: 5%; $E_{s}$: 116%;
Tekinalp et al. [45]	Short carbon fibre/ABS	30 wt%	Tensile	ASTM D638	$\sigma_{t}$: 115%; $E_{t}$: 700%;
Tian et al. [22]	Continuous carbon fibre/PLA	9 vol%	Tensile; Flexural	GB/T 1447; GB/T 1449	$\sigma_{t}$: 313%; $E_{t}$: 390%; $\sigma_{f}$: 260%;$\;E_{f}$: 230%
Hinchcliffe et al. [46]	Continuous flax fibre/PLA	NR	Tensile	ASTM D638	$\sigma_{t}$: 116%; $E_{t}$: 62%; $\sigma_{f}$: 14%;$\;E_{f}$: 10%
Matsuzaki et al. [47]	Continuous carbon fibre/PLA	6 vol%	Tensile	JIS K 7162	$\sigma_{t}$: 363%; $E_{t}$: 400%
Shofner et al. [29]	Nanocarbon fibre/ABS	10 wt%	Tensile	ASTM D638	$\sigma_{t}$: 39%; $E_{t}$: 40%
Dutra et al. [48]	Continuous carbon fibre/polyamide	30 vol%	Tensile	ASTM D3039	$E_{t}$: 894%
Caminero et al. [49]	Continuous carbon fibre/polyamide; Continuous Kevlar fibre/polyamide; Continuous glass fibre/polyamide	50 wt%	Impact	ASTM D6110	Impact resistance: Continuous carbon fibre/polyamide: 181%; Continuous Kevlar fibre/polyamide/: 513%; Continuous glass fibre/polyamide: 1225%
Naranjo-Lozada et al. [50]	Short carbon fibre/polyamide; Continuous carbon fibre/polyamide	NR; 50 wt%	Tensile	ASTM D638	Short fibre: $\sigma_{t}$: 46%; $E_{t}$: 115% Continuous fibre: $\sigma_{t}$: 2826%; $E_{t}$: 3848%
Dickson et al. [51]	Continuous carbon fibre/polyamide; Continuous Kevlar fibre/polyamide; Continuous glass fibre/polyamide	8–11 vol%	Tensile; Flexural	ASTM D3039; ASTM D7264	Continuous carbon fibre/polyamide: $\sigma_{t}$: 254%; $E_{t}$: 1358%; $\sigma_{f}$: 260%;$\;E_{f}$: 1128% Continuous Kevlar fibre/polyamide: $\sigma_{t}$: 169%; $E_{t}$: 725%; $\sigma_{f}$: 200%;$\;E_{f}$: 527% Continuous glass fibre/polyamide: $\sigma_{t}$: 238%; $E_{t}$: 608%; $\sigma_{f}$: 369%;$\;E_{f}$: 297%

* $\sigma_{t}$, $\sigma_{f}$, $\sigma_{c}$, $\sigma_{s}$ refer to the improvements of tensile strength, flexural strength, compressive strength and shear strength, compared to printed polymer matrix, respectively. $E_{t}$, $E_{f}$, $E_{c}$, $E_{s}$ refer to the improvements of tensile modulus, flexural modulus, compressive modulus and shear modulus, compared to printed polymer matrix, respectively.

**Table 3:** The crystallinity and melting temperature of filaments, fabricated polyamide and SFRN samples.

			Polyamide		SFRN	
Raster Pattern	Layer Thickness (mm)		T_m_ (°C)	Crystallinity (%)	T_m_ (°C)	Crystallinity (%)
[0, 90]	0.1	1st heating	200	18	199	17
		2nd heating	202	20	200	18
	0.125	1st heating	200	16	200	17
		2nd heating	201	17	200	17
	0.2	1st heating	199	16	199	17
		2nd heating	200	19	200	18
[+45, −45]	0.1	1st heating	199	19	197	17
		2nd heating	201	20	200	20
	0.125	1st heating	200	17	197	16
		2nd heating	201	18	200	18
	0.2	1st heating	201	18	197	18
		2nd heating	202	19	199	20
Filament		1st heating	200	35	199	21
		2nd heating	201	19	201	20
Injection-moulded		1st heating	199	18	199	18
		2nd heating	198	20	199	20

**Table 4:** The tensile properties of the printed and injection-moulded samples.

			Polyamide		SFRN	
Thick (mm)	Layer Thickness (mm)	Raster Pattern	Yield Stress (MPa)	Tensile Modulus (GPa)	Yield Stress (MPa)	Tensile Modulus (GPa)
2	0.1	[0, 90]	32.5 ± 0.6	1.19 ± 0.04	51.5 ± 3.6	3.25 ± 0.10
		[+45, −45]	30.3 ± 0.2	1.11 ± 0.05	53.7 ± 1.2	3.00 ± 0.08
	0.125	[0, 90]	32.3 ± 0.7	1.17 ± 0.02	53.8 ± 1.6	3.57 ± 0.19
		[+45, −45]	30.4 ± 0.5	1.16 ± 0.10	57.3 ± 2.1	3.16 ± 0.08
	0.2	[0, 90]	33.8 ± 0.4	1.21 ± 0.07	55.2 ± 1.3	4.08 ± 0.21
		[+45, −45]	31.2 ± 0.9	1.14 ± 0.07	57.5 ± 2.6	3.56 ± 0.10
3	0.1	[0, 90]	33.5 ± 1.1	1.12 ± 0.06	54.5 ± 2.6	3.88 ± 0.18
		[+45, −45]	34.3 ± 0.9	1.18 ± 0.11	56.8 ± 1.6	3.66 ± 0.17
	0.125	[0, 90]	33.2 ± 0.8	1.13 ± 0.09	54.7 ± 1.9	4.03 ± 0.22
		[+45, −45]	34.0 ± 0.9	1.12 ± 0.09	56.7 ± 2.1	3.82 ± 0.24
	0.2	[0, 90]	33.3 ± 1.5	1.17 ± 0.07	56.8 ± 2.4	4.57 ± 0.27
		[+45, −45]	34.1 ± 0.4	1.16 ± 0.11	58.5 ± 1.7	4.11 ± 0.21
	Injection-moulded	-	37.6 ± 0.4	1.43 ± 0.22	78.1 ± 3.5	6.67 ± 0.22
4	0.1	[0, 90]	34.3 ± 0.5	1.19 ± 0.05	55.7 ± 3.7	4.25 ± 0.19
		[+45, −45]	32.9 ± 0.4	1.19 ± 0.09	58.8 ± 1.9	4.13 ± 0.30
	0.125	[0, 90]	32.9 ± 0.7	1.20 ± 0.03	56.6 ± 1.7	4.48 ± 0.25
		[+45, −45]	32.5 ± 0.4	1.11 ± 0.07	60.1 ± 2.4	4.25 ± 0.24
	0.2	[0, 90]	32.9 ± 0.7	1.16 ± 0.08	59.5 ± 3.7	4.90 ± 0.23
		[+45, −45]	34.1 ± 0.4	1.21 ± 0.08	59.8 ± 4.2	4.57 ± 0.21

**Table 5:** The interface density of the printed SFRN samples.

Sample Thick ($T_{S}$, mm)	Layer Thickness ($T_{L}$, mm)	Number of Interfaces $\left(T_{S}/T_{L}-1\right)$	Interface Density $\left(\frac{1}{T_{L}}-\frac{1}{T_{S}}\right)$, /mm)	Linear Correlation [0, 90]	Linear Correlation [+45, −45]
2	0.1	20	9.50	0.999	
	0.125	16	7.50		0.983
	0.2	10	4.50		
3	0.1	30	9.67	0.960	
	0.125	24	7.67		0.998
	0.2	15	4.67		
4	0.1	40	9.75	0.997	
	0.125	32	7.75		0.980
	0.2	20	4.75		

## Data Availability

The data presented in this study are available on request from the corresponding author.

## References

[1] Brenken B., Barocio E., Favaloro A., Kunc V., Pipes R.B. (2018). Fused Filament Fabrication of Fiber-Reinforced Polymers: A Review. Addit. Manuf..

[2] Joshi S.C., Sheikh A.A. (2015). 3D Printing in Aerospace and Its Long-Term Sustainability. Virtual Phys. Prototyp..

[3] Shahrubudin N., Lee T.C., Ramlan R. (2019). An Overview on 3D Printing Technology: Technological, Materials, and Applications. Procedia Manuf..

[4] Yao X., Luan C., Zhang D., Lan L., Fu J. (2017). Evaluation of Carbon Fiber-Embedded 3D Printed Structures for Strengthening and Structural-Health Monitoring. Mater. Des..

[5] Chacón J.M., Caminero M.A., García-Plaza E., Núñez P.J. (2017). Additive Manufacturing of PLA Structures Using Fused Deposition Modelling: Effect of Process Parameters on Mechanical Properties and Their Optimal Selection. Mater. Des..

[6] Yu N., Sun X., Wang Z., Zhang D., Li J. (2021). Effects of Auxiliary Heat on the Interlayer Bonds and Mechanical Performance of Polylactide Manufactured through Fused Deposition Modeling. Polym. Test..

[7] Cicala G., Giordano D., Tosto C., Filippone G., Recca A., Blanco I. (2018). Polylactide (PLA) Filaments a Biobased Solution for Additive Manufacturing: Correlating Rheology and Thermomechanical Properties with Printing Quality. Materials.

[8] Tymrak B.M., Kreiger M., Pearce J.M. (2014). Mechanical Properties of Components Fabricated with Open-Source 3-D Printers under Realistic Environmental Conditions. Mater. Des..

[9] D’Amico A.A., Debaie A., Peterson A.M. (2017). Effect of Layer Thickness on Irreversible Thermal Expansion and Interlayer Strength in Fused Deposition Modeling. Rapid Prototyp. J..

[10] Ziemian S., Okwara M., Ziemian C.W. (2015). Tensile and Fatigue Behavior of Layered Acrylonitrile Butadiene Styrene. Rapid Prototyp. J..

[11] Lee J., Huang A. (2013). Fatigue Analysis of FDM Materials. Rapid Prototyp. J..

[12] Zhou Y.G., Zou J.R., Wu H.H., Xu B.P. (2020). Balance between Bonding and Deposition during Fused Deposition Modeling of Polycarbonate and Acrylonitrile-Butadiene-Styrene Composites. Polym. Compos..

[13] Salazar-Martín A.G., Pérez M.A., García-Granada A.A., Reyes G., Puigoriol-Forcada J.M. (2018). A Study of Creep in Polycarbonate Fused Deposition Modelling Parts. Mater. Des..

[14] Puigoriol-Forcada J.M., Alsina A., Salazar-Martín A.G., Gomez-Gras G., Pérez M.A. (2018). Flexural Fatigue Properties of Polycarbonate Fused-Deposition Modelling Specimens. Mater. Des..

[15] Toro E.V.D., Sobrino J.C., Mart A., Ayll J. (2020). Investigation of a Short Carbon Fibre-Reinforced Polyamide and Comparison of Two Manufacturing Processes: Fused Deposition Modelling (FDM) and Polymer Injection Moulding (PIM). Materials.

[16] Zhang X., Fan W., Liu T. (2020). Fused Deposition Modeling 3D Printing of Polyamide-Based Composites and Its Applications. Compos. Commun..

[17] Gao X., Zhang D., Wen X., Qi S., Su Y., Dong X. (2019). Fused Deposition Modeling with Polyamide 1012. Rapid Prototyp. J..

[18] Song Y., Li Y., Song W., Yee K., Lee K.Y., Tagarielli V.L. (2017). Measurements of the Mechanical Response of Unidirectional 3D-Printed PLA. Mater. Des..

[19] Ning F., Cong W., Qiu J., Wei J., Wang S. (2015). Additive Manufacturing of Carbon Fiber Reinforced Thermoplastic Composites Using Fused Deposition Modeling. Compos. Part B Eng..

[20] Love L.J., Kunc V., Rios O., Duty C.E., Elliott A.M., Post B.K., Smith R.J., Blue C.A. (2014). The Importance of Carbon Fiber to Polymer Additive Manufacturing. J. Mater. Res..

[21] Omuro R., Ueda M., Matsuzaki R., Todoroki A., Hirano Y. Three-Dimensional Printing of Continuous Carbon Fiber Reinforced Thermoplastics By in-Nozzle Impregnation With Compaction Roller. Proceedings of the 21st International Conference on Composite Materials.

[22] Tian X., Liu T., Wang Q., Dilmurat A., Li D., Ziegmann G. (2017). Recycling and Remanufacturing of 3D Printed Continuous Carbon Fiber Reinforced PLA Composites. J. Clean. Prod..

[23] Van Der Klift F., Koga Y., Todoroki A., Ueda M., Hirano Y., Matsuzaki R. (2016). 3D Printing of Continuous Carbon Fibre Reinforced Thermo-Plastic (CFRTP) Tensile Test Specimens. Open J. Compos. Mater..

[24] Cantrell J.T., Rohde S., Damiani D., Gurnani R., DiSandro L., Anton J., Young A., Jerez A., Steinbach D., Kroese C. (2017). Experimental Characterization of the Mechanical Properties of 3D-Printed ABS and Polycarbonate Parts. Rapid Prototyp. J..

[25] McLouth T.D., Severino J.V., Adams P.M., Patel D.N., Zaldivar R.J. (2017). The Impact of Print Orientation and Raster Pattern on Fracture Toughness in Additively Manufactured ABS. Addit. Manuf..

[26] Rahmatabadi D., Ghasemi I., Baniassadi M., Abrinia K., Baghani M. (2022). 3D Printing of PLA-TPU with Different Component Ratios: Fracture Toughness, Mechanical Properties, and Morphology. J. Mater. Res. Technol..

[27] Rahmatabadi D., Soltanmohammadi K., Aberoumand M., Soleyman E., Ghasemi I., Baniassadi M., Abrinia K., Bodaghi M., Baghani M. (2022). Development of Pure Poly Vinyl Chloride (PVC) with Excellent 3D Printability and Macro- and Micro-Structural Properties. Macromol. Mater. Eng..

[28] Moradi M., Aminzadeh A., Rahmatabadi D., Rasouli S.A. (2021). Statistical and Experimental Analysis of Process Parameters of 3D Nylon Printed Parts by Fused Deposition Modeling: Response Surface Modeling and Optimization. J. Mater. Eng. Perform..

[29] Shofner M.L., Lozano K., Rodrı F.J. (2003). Nanofiber-Reinforced Polymers Prepared by Fused Deposition Modeling. J. Appl. Polym. Sci..

[30] Patanwala H.S., Hong D., Vora S.R., Bognet B., Ma A.W.K. (2018). The Microstructure and Mechanical Properties of 3D Printed Carbon Nanotube-Polylactic Acid Composites. Polym. Compos..

[31] Przekop R.E., Kujawa M., Pawlak W., Dobrosielska M., Sztorch B., Wieleba W. (2020). Graphite Modified Polylactide (PLA) for 3D Printed (FDM/FFF) Sliding Elements. Polymers.

[32] Jia Y., He H., Geng Y., Huang B., Peng X. (2017). High Through-Plane Thermal Conductivity of Polymer Based Product with Vertical Alignment of Graphite Flakes Achieved via 3D Printing. Compos. Sci. Technol..

[33] Ferreira R.T.L., Amatte I.C., Dutra T.A., Bürger D. (2017). Experimental Characterization and Micrography of 3D Printed PLA and PLA Reinforced with Short Carbon Fibers. Compos. Part B Eng..

[34] Yang Z., Yang Z., Chen H., Yan W. (2022). 3D Printing of Short Fiber Reinforced Composites via Material Extrusion: Fiber Breakage. Addit. Manuf..

[35] Ma S., Yang H., Zhao S., He P., Zhang Z., Duan X., Yang Z., Jia D., Zhou Y. (2021). 3D-Printing of Architectured Short Carbon Fiber-Geopolymer Composite. Compos. Part B Eng..

[36] Zhong W., Li F., Zhang Z., Song L., Li Z. (2001). Short Fiber Reinforced Composites for Fused Deposition Modeling. Mater. Sci. Eng. A.

[37] Sodeifian G., Ghaseminejad S., Yousefi A.A. (2019). Preparation of Polypropylene/Short Glass Fiber Composite as Fused Deposition Modeling (FDM) Filament. Results Phys..

[38] Ghabezi P., Flanagan T., Harrison N. (2022). Short Basalt Fibre Reinforced Recycled Polypropylene Filaments for 3D Printing. Mater. Lett..

[39] Sang L., Han S., Li Z., Yang X., Hou W. (2019). Development of Short Basalt Fiber Reinforced Polylactide Composites and Their Feasible Evaluation for 3D Printing Applications. Compos. Part B Eng..

[40] Hao W., Liu Y., Zhou H., Chen H., Fang D. (2018). Preparation and Characterization of 3D Printed Continuous Carbon Fiber Reinforced Thermosetting Composites. Polym. Test..

[41] Le Duigou A., Castro M., Bevan R., Martin N. (2016). 3D Printing of Wood Fibre Biocomposites: From Mechanical to Actuation Functionality. Mater. Des..

[42] Galos J., Hu Y., Ravindran A.R., Ladani R.B., Mouritz A.P. (2021). Electrical Properties of 3D Printed Continuous Carbon Fibre Composites Made Using the FDM Process. Compos. Part A Appl. Sci. Manuf..

[43] Hu Y., Ladani R.B., Brandt M., Li Y., Mouritz A.P. (2021). Carbon Fibre Damage during 3D Printing of Polymer Matrix Laminates Using the FDM Process. Mater. Des..

[44] Mahajan C., Cormier D. 3D Printing of Carbon Fiber Composites with Preferentially Aligned Fibers. Proceedings of the 2015 Industrial and Systems Engineering Research Conference.

[45] Tekinalp H.L., Kunc V., Velez-Garcia G.M., Duty C.E., Love L.J., Naskar A.K., Blue C.A., Ozcan S. (2014). Highly Oriented Carbon Fiber-Polymer Composites via Additive Manufacturing. Compos. Sci. Technol..

[46] Hinchcliffe S.A., Hess K.M., Srubar W.V. (2016). Experimental and Theoretical Investigation of Prestressed Natural Fiber-Reinforced Polylactic Acid (PLA) Composite Materials. Compos. Part B Eng..

[47] Matsuzaki R., Ueda M., Namiki M., Jeong T.K., Asahara H., Horiguchi K., Nakamura T., Todoroki A., Hirano Y. (2016). Three-Dimensional Printing of Continuous-Fiber Composites by in-Nozzle Impregnation. Sci. Rep..

[48] Dutra T.A., Ferreira R.T.L., Resende H.B., Guimarães A. (2019). Mechanical Characterization and Asymptotic Homogenization of 3D-Printed Continuous Carbon Fiber-Reinforced Thermoplastic. J. Braz. Soc. Mech. Sci. Eng..

[49] Caminero M.A., Chacón J.M., García-Moreno I., Rodríguez G.P. (2018). Impact Damage Resistance of 3D Printed Continuous Fibre Reinforced Thermoplastic Composites Using Fused Deposition Modelling. Compos. Part B Eng..

[50] Naranjo-Lozada J., Ahuett-Garza H., Orta-Castañón P., Verbeeten W.M.H., Sáiz-González D. (2019). Tensile Properties and Failure Behavior of Chopped and Continuous Carbon Fiber Composites Produced by Additive Manufacturing. Addit. Manuf..

[51] Dickson A.N., Barry J.N., McDonnell K.A., Dowling D.P. (2017). Fabrication of Continuous Carbon, Glass and Kevlar Fibre Reinforced Polymer Composites Using Additive Manufacturing. Addit. Manuf..

[52] Zhuo P., Li S., Ashcroft I., Jones A., Pu J., Science C., Division T. 3D Printing of Continuous Fibre Reinforced Thermoplastic Composite. Proceedings of the 21st International Conference on Composite Materials.

[53] Griffini G., Invernizzi M., Levi M., Natale G., Postiglione G., Turri S. (2016). 3D-Printable CFR Polymer Composites with Dual-Cure Sequential IPNs. Polymer.

[54] Kariz M., Sernek M., Obućina M., Kuzman M.K. (2018). Effect of Wood Content in FDM Filament on Properties of 3D Printed Parts. Mater. Today Commun..

[55] Compton B.G., Lewis J.A. (2014). 3D-Printing of Lightweight Cellular Composites. Adv. Mater..

[56] Blok L.G., Longana M.L., Yu H., Woods B.K.S. (2018). An Investigation into 3D Printing of Fibre Reinforced Thermoplastic Composites. Addit. Manuf..

[57] Zhang W., Cotton C., Sun J., Heider D., Gu B., Sun B., Chou T.W. (2018). Interfacial Bonding Strength of Short Carbon Fiber/Acrylonitrile-Butadiene-Styrene Composites Fabricated by Fused Deposition Modeling. Compos. Part B Eng..

[58] Christensen K., Davis B., Jin Y., Huang Y. (2018). Effects of Printing-Induced Interfaces on Localized Strain within 3D Printed Hydrogel Structures. Mater. Sci. Eng. C.

[59] Bellehumeur C., Li L., Sun Q., Gu P. (2004). Modeling of Bond Formation between Polymer Filaments in the Fused Deposition Modeling Process. J. Manuf. Process..

[60] Gurrala P.K., Regalla S.P. (2014). Part Strength Evolution with Bonding between Filaments in Fused Deposition Modelling: This Paper Studies How Coalescence of Filaments Contributes to the Strength of Final FDM Part. Virtual Phys. Prototyp..

[61] Li L., Sun Q., Bellehumeur C., Gu P. (2019). Investigation of Bond Formation in FDM Process Investigation of Bond Formation in FDM Process. Trans. N. Am. Manuf. Res. Inst. SME.

[62] Everett H. Markforged Merges with One to Go Public as a $2.1 Billion 3D Printing Business. https://3dprintingindustry.com/news/markforged-merges-with-one-to-go-public-as-a-2-1-billion-3d-printing-business-185077.

[63] Sommacal S., Matschinski A., Drechsler K., Compston P. (2021). Characterisation of Void and Fiber Distribution in 3D Printed Carbon-Fiber/PEEK Using X-Ray Computed Tomography. Compos. Part A Appl. Sci. Manuf..

[64] MarkForged FFF Nylon Filament, Safety Data Sheet. https://markforged.com/materials/plastics/nylon.

[65] Millot C., Fillot L.A., Lame O., Sotta P., Seguela R. (2015). Assessment of Polyamide-6 Crystallinity by DSC: Temperature Dependence of the Melting Enthalpy. J. Therm. Anal. Calorim..

[66] (2015). ASTM International D638-14 Standard Test Method for Tensile Properties of Plastics.

[67] Cox H.L. (1952). The Elasticity and Strength of Paper and Other Fibrous Materials. Br. J. Appl. Phys..

[68] Krenchel H. (1964). Fibre Reinforcement; Theoretical and Practical Investigations of the Elasticity and Strength of Fibre-Reinforced Materials.

[69] O’Regan D.F., Akay M., Meenan B. (1999). A Comparison of Young’s Modulus Predictions in Fibre-Reinforced-Polyamide Injection Mouldings. Compos. Sci. Technol..

[70] Parodi E., Govaert L.E., Peters G.W.M. (2017). Glass Transition Temperature versus Structure of Polyamide 6: A Flash-DSC Study. Thermochim. Acta.

[71] Sun Q., Rizvi G.M., Bellehumeur C.T., Gu P. (2008). Effect of Processing Conditions on the Bonding Quality of FDM Polymer Filaments. Rapid Prototyp. J..

[72] Sood A.K., Ohdar R.K., Mahapatra S.S. (2010). Parametric Appraisal of Mechanical Property of Fused Deposition Modelling Processed Parts. Mater. Des..

[73] Sauer M.J. (2018). Evaluation of the Mechanical Properties of 3D Printed Carbon Fiber Composites.

[74] Performance Composites Ltd. (2009). Mechanical Properties of Carbon Fibre Composite Materials, Fibre/Epoxy Resin (120 °C Cure).

[75] Greaves G.N., Greer A.L., Lakes R.S., Rouxel T. (2011). Poisson’s Ratio and Modern Materials. Nat. Mater..

[76] Nairn J.A., Mendels D.A. (2001). On the Use of Planar Shear-Lag Methods for Stress-Transfer Analysis of Multilayered Composites. Mech. Mater..

[77] Carraro P.A., Quaresimin M. (2015). A Stiffness Degradation Model for Cracked Multidirectional Laminates with Cracks in Multiple Layers. Int. J. Solids Struct..

[78] Kashtalyan M., Soutis C. (2006). Modelling Off-Axis Ply Matrix Cracking in Continuous Fibre-Reinforced Polymer Matrix Composite Laminates. J. Mater. Sci..

[79] McCartney L.N. (1992). Theory of Stress Transfer in a 0°–90°–0° Cross-Ply Laminate Containing a Parallel Array of Transverse Cracks. J. Mech. Phys. Solids.

[80] Gudmundson P., Weilin Z. (1993). An Analytic Model for Thermoelastic Properties of Composite Laminates Containing Transverse Matrix Cracks. Int. J. Solids Struct..

